# Consumers acceptance of new food ingredients from the food industry’s by-products—a focus group study

**DOI:** 10.3389/fnut.2025.1509833

**Published:** 2025-03-19

**Authors:** Sophie Scheibenzuber, Emilia Pucci, Ombretta Presenti, Giacomo Serafini, Chiara Nobili, Claudia Zoani, Denisa Eglantina Duta, Adriana Laura Mihai, Gabriela Daniela Criveanu-Stamatie, Nastasia Belc, Eva Falch, Turid Rustad, Michael Rychlik

**Affiliations:** ^1^Department of Life Science Engineering, School of Life Sciences, Technical University of Munich, Freising, Germany; ^2^Department for Sustainability, Sustainable AgriFood Systems Division, Italian National Agency for New Technologies, Energy and Sustainable Economic Development (ENEA), Rome, Italy; ^3^National Research and Development Institute for Food Bioresources, Bucharest, Romania; ^4^Department of Biotechnology and Food Science, Norwegian University of Science and Technology, Trondheim, Norway

**Keywords:** focus group study, food products, new ingredients, by-products, consumer acceptance

## Abstract

Food industry by-products can be valorized by extracting various nutritional components, like proteins, dietary fiber or other bioactive compounds, depending on the type of by-products. By adding these to new or already existing food products, the consumers’ health and wellbeing may increase due to a more nutritional diet while at the same time increasing the sustainability of the food chain. However, for a successful market implementation of products containing ingredients from by-products, the consumer perception and consumer acceptance are one of the most important aspects that need to be considered. Therefore, focus group studies were organized in four European countries, namely Italy, Germany, Romania and Norway, to investigate the perception, acceptance and willingness of the general population (ages 18–60) to buy these new products. When possible, individual purchasing and consumption trends were analyzed as well to get a deeper insight into the decision-making processes during grocery shopping. In summary, the acceptance of new food ingredients from by-products was high in all four studied countries, and most participants were interested in enriched bakery, meat or dairy products. The main reason for the interest in these new products were health benefits due to an increased nutrient uptake, and to contribute to reducing food waste. However, participants were afraid of new food allergies and intolerances as well as increased concentrations of food contaminants like pesticides or mycotoxins, which makes food safety an important point to consider before developing new products.

## 1 Introduction

As the world’s population is expected to be constantly growing over the next decades, the need for a healthy, nutritional, and sustainable food supply is rising as well, which poses a challenge to both agriculture and food producers. However, many projections are centered on increasing food production by at least 70% rather than also focusing on reducing food loss and food waste (1, 2).

According to the FAO, the amount of consumable food that gets lost or wasted worldwide during the food production and in retail, restaurants or at home, sums up to about 1.3 billion tons per year (3, 4). Further projections estimate that the total food loss and waste will reach 2.1 billion tons per year by 2030 (5). In Europe, with 88 million tons of food waste being produced every year, 31 million tons of food waste would need to be reduced each year to meet the United Nations sustainable development goal No. 12 “Responsible Consumption and Production,” and in particular its target 12–3, which aims to halve the per capita global food waste and to reduce food losses along production and supply chains. Consequently, new innovative ways have to be found to prevent or reuse and recycle the currently available food waste (6–8).

Besides avoidable food waste, there is also a lot of product-specific food waste, the so-called food by-products. Large proportions of this industrial food waste are unavoidable, as the amount and kind of waste can scarcely be altered if the quality of the finished product is to remain consistent (2, 9). Some examples of the most common food by-products are spent grains from beer production, whey from the dairy industry, oilseed press cakes from the oil industry and poultry waste (e.g., bones, skins, feathers etc.) (52). Despite their huge potential as valuable source for nutrients, these by-products are often wasted or used as low-quality animal feed or for biogas production (10). By applying valorization techniques, these waste products can serve as raw material sources for different nutritional components like dietary fiber, proteins, polyphenols, antioxidants and other bioactive compounds, which can be added to food products in order to increase their nutritional value (2, 10, 11). This addition of beneficial ingredients to commonly used food products (e.g., bread or pasta) is also commonly known as “upcycled food” (12).

However, before food producers invest in valorization techniques, the consumer perception and acceptance of new food products from by-products are some of the most important aspects that need to be considered for a successful market implementation. One problem, for example, that was already identified by Aschemann-Witzel and Stangherlin (13) is that the terms “by-product” and “food waste” are often treated equally, which brings a negative association to by-products from food side-streams as it is often seen as useless, disgusting and unsafe.

To get a better insight into consumer behavior, focus group studies—as well as one-to-one interviews—are currently the state-of-the-art tools for the development of reliable and valid consumer surveys (14). The main purpose of these kinds of studies is to understand the behavior of the chosen participant group, to gain insight into the individual motivation and judgments, to understand the ways the participants form their views and opinions, and to learn more about how they make decisions (15, 16). Focus group interviews were also previously described as “carefully planned discussion designed to obtain perceptions on a defined area of interest in a permissive, non-threatening environment” (14). One main advantage of a study like this is that group interactions can stimulate the debate on a certain topic and encourage participants to explore and clarify their views (14). Focus groups ideally consist of between 6 and 10 participants, as this size, on the one hand, is big enough to give the participants a sense of safety, and, on the other hand, is small enough to maintain a coherent course of discussion, and to let each participant express his or her opinion on a certain topic (16). As the participants are establishing the relevance of the topic under discussion, a careful selection of the study participants is important, as some topics might be irrelevant for certain parts of society (15).

The main objective of this study was to investigate the acceptance of foods with ingredients from food by-products. For this purpose, we conducted a focus group case study in four European countries, namely Germany, Italy, Romania, and Norway to get a better insight into the overall perception and acceptance of such products. In addition, the local purchasing and consumption trends, and the participants’ decision-making processes during the purchase were analyzed as well where possible.

## 2 Materials and methods

In total, four focus groups interviews were conducted, namely one in each of the four countries (Germany, Italy, Romania, and Norway) (17). All four groups represented the “general population,” i.e., persons aged 18–60, and comprised six (Germany, Italy, Romania) or seven (Norway) participants.

### 2.1 Study background

The background of the study was to get further insight into the general knowledge on food by-products and their valorization as well as consumer preferences and their individual acceptance regarding new food developments with ingredients from by-products, such as dairy, oilseeds, brewery, meat (poultry) and the prickly pear cactus.

Factors that were taken into consideration for choosing the mentioned by-products (except the prickly pear cactus) were:

- Amounts generated (in Europe and worldwide).
- Production distribution in different countries (= by-products should be available in all four participating countries).
- Composition of by-products (= valuable compounds for valorization).
- Safety aspects.

The prickly pear cactus was chosen due to its properties as “future crop” due to its potential to grow in dry regions and its beneficial nutritional composition. While it is mainly cultivated in South America and Afrika, it currently also grows in Italy, Portugal, and Spain (18).

### 2.2 Participant selection

In each country, participants were recruited through social media, flyers, e-mail newsletters, personal contacts, and further public announcements.

Interested persons were asked to fill out a questionnaire (see Supplementary material), which was evaluated by the national research teams to see if the candidates were suitable for the study. The following screening criteria were applied during participant recruitment and participant selection:

- The person must be the main decision maker about grocery/food shopping or share this responsibility equally with another household member.
- Participants do not work in sensitive industry (food industry, marketing, etc.)
- Participants did not participate in food-related research recently.

The final choice of participants for the focus groups was made under the following additional points of view:

- Each group should be heterogeneous in terms of their animal and plant-based food consumption, and of people who consume meat, avoid meat, and are vegetarians/vegans.
- Each group should be of mixed gender.
- Each group should have a good spread of age.

In the end, the focus groups were composed as shown in Table 1.

**Table 1:** Composition of the four focus groups.

Country	Gender	Age	Education
Germany	3 male, 3 female	1 person between 20 and 30, 3 people between 30 and 40, 1 person between 40 and 50 and 1 person between 50 and 60	3 participants: higher education 1 participant: university student 2 participants: high school diploma
Italy	3 male, 3 female	2 people between 20 and 30, 2 people between 30 and 40, 2 person between 40 and 50	1 participant: university student 5 participants: higher education
Romania	3 male, 3 female	1 person between 20 and 30, 5 people between 30 and 40	All participants with higher education
Norway	2 male, 5 female	3 people between 20 and 30, 3 people between 30 and 40, 1 person between 50 and 60	5 participants: higher education 2 participants: high school diploma

### 2.3 Planning of the focus group interviews

A methodology for focus groups was developed between the four partner countries to obtain qualitative information from consumers in the selected countries.

Each focus group interview was planned to be about 120 min long. The exact process and interview questions were discussed and coordinated beforehand between the four executing countries to guarantee comparable results in all four interviews. The resulting complete “moderators’ protocol” can be found in Supplementary material.

In short, the focus group discussion was split into three thematic parts: Exploration of general knowledge on food by-products (Stage 1, 25–35 min), Exploration of food products with by-products (Stage 2, 20–30 min) and Exploration of purchases and consumption trends & decision-making processes (Stage 3, 45–55 min). To evaluate the decision-making process, the participants received an explanation and introduction to the idea of food products with/from by-products. More specifically, participants were given a description of the general idea of enriching products using by-products from different food production chains, such as: dairy, oilseeds, brewery, meat (poultry) and prickly pear cactus.

### 2.4 Conduction of the focus group interviews

The interviews were conducted both in presence (Italy and Norway) and online (Germany and Romania), due to still ongoing Covid prevention measures in some countries. All focus groups were held in the national language to allow an open discussion without a language barrier.

At the beginning, participants were welcomed to the study and the moderators introduced themselves, the study background and the study procedure as well as the timeline. After a brief introduction from each participant, the discussion was started as described in the moderators’ protocol (see Supplementary material).

The focus groups were done in a semi-structured way, allowing the moderator and the participants of the group to raise unknown issues or other than those already assigned in the agenda. Via this qualitative methodology, the moderator was allowed to ask the participants about the comparisons between their views and experiences, facilitating a better understanding of why the participants agree or disagree.

### 2.5 Data analysis

All focus group interviews were recorded by the moderator for subsequent data analysis according to standard procedures. During the interview, an assistant was present as well to take notes and write down a summary of the main results. After the conclusion of the interview, the moderator and assistant had a debrief, where initial impressions of the discussion as well as highlights, issues, or immediate conclusions were drawn. Afterwards, the recorded data was transcribed and cleaned from personal information that could lead back to the participants’ identity. During transcription, a constant content analysis for themes and categories of responses was made. In addition, a standard procedure for data analysis and some questions were specified by the representatives of all four countries before starting data analysis to allow a comparable data set (e.g., what patterns emerge, what are common themes, which new questions arise), which is in accordance to the procedure described by Braun and Clarke (19). National data were analyzed by at least two independent people, while one additional person was in charge of re-analyzing the data collected in all four countries. For data analysis, all transcripts were translated into English by the national representatives.

These categories served as basis for reporting and comparing the results from each country.

### 2.6 Ethical issues

During the participant recruiting process, each participant received adequate information about the study, data collection and data handling according to the General Data Protection Regulation. Their consent was mandatory for participating in the focus group interviews.

Before the start of the interviews, all participants were informed of the audio-taping during the study, and the data processing was explained. It was also mentioned that the contents of the interviews and the research data are treated as strictly confidential and that they can quit at any time.

To guarantee confidentiality, each participant was assigned a number or pseudonym for organizational purposes before the study.

## 3 Results

As data were collected in two different forms (through questionnaires before and during the third session of the focus group study, and through open discussions within the focus groups), results will be presented accordingly. When questionnaires were used (sections 3.1 consumption trends and 3.4 exploration of decision making processes), all participants filled out the same tasks/questions, which were then compared between the different countries. To present the results from the open discussion, the first two stages of the focus group interviews described in section 2.3 were analyzed for themes and summarized in subchapters 3.2 and 3.3. However, as the sections of the focus group discussions were already thematically organized, in most cases the themes were already given by the respective topic of the section.

### 3.1 Current consumption trends observed within the focus groups

Before being selected to participate in the focus group discussion, each participant filled out a pre-questionnaire (see Supplementary material) in which the participants were asked about their current consumption trends. In detail, they were asked about the frequency (once a week or more, 2–3 times a month, once a month, rarely than once per month, or never) of their consumption of the following food items: Whey products (cheeses, butter, protein concentrate, whey powder etc.), whole cereals, oleaginous seeds, bread from different cereals/oleaginous seeds, foods containing prickly pear cactus by-products or ingredients extracted from prickly pear cactus by-products, yogurts with increased protein content, milk, bread with increased fiber content (either whole wheat bread or bread with added fibrous ingredients), poultry by-products or ingredients extracted from them.

**Figure 1:**
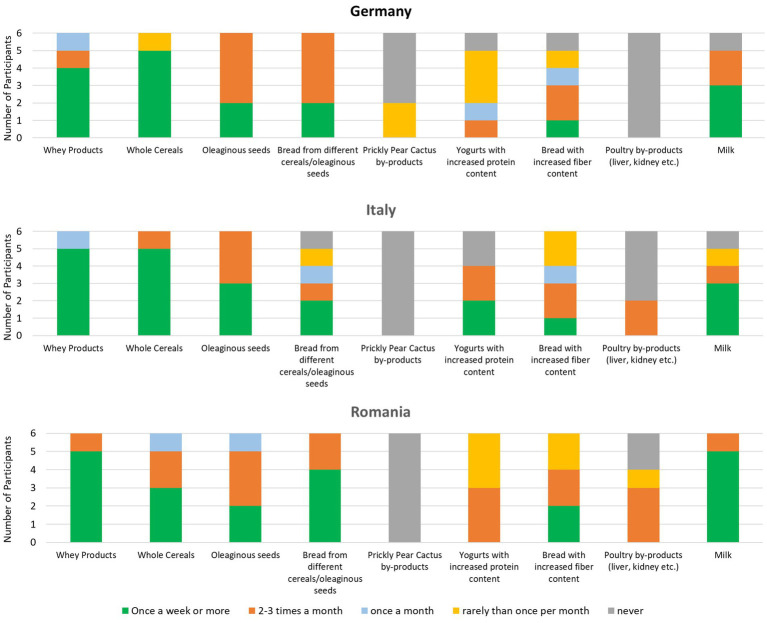
Current consumption trends of focus group participants in Germany, Italy, and Romania. No detailed data was collected in Norway.

A summary of the obtained data can be found in Figure 1.

The consumption trends within the different focus groups in the three studied countries were comparable for many products especially when looking at whey products, whole cereals and oleaginous seeds. Bread from different cereals is consumed most often in Germany, while bread with increased fiber content is consumed by almost all participants from time to time. While no participant from Italy and Romania consumed the prickly pear cactus, two participants from Germany stated that they eat it “rarely than once per month.” However, when asked, they clarified that they have consumed it on vacation outside Germany. With protein-enriched yogurts and milk the consumption pattern was quite variable, but most participants of the three groups consume those products at least from time to time. The most controverse results were obtained with poultry by-products, which were not consumed at all by German participants while two participants from Italy consume them 2–3 times a month. In Romania, only two participants never consume poultry by-products, while 4 people have consumed them before or eat them on a regular basis.

In Norway, participants were asked in general if they consumed the above-mentioned food products regularly to which they responded that they all consumed milk/whey products regularly as well as whole grain cereals/whole grain bread. By regularly they would mean once a week or more or 2–3 times per month. No one of the Norwegian focus group study consumed the prickly pear cactus.

### 3.2 Stage 1: exploration of the general knowledge on food by-products

**Figure 2:**
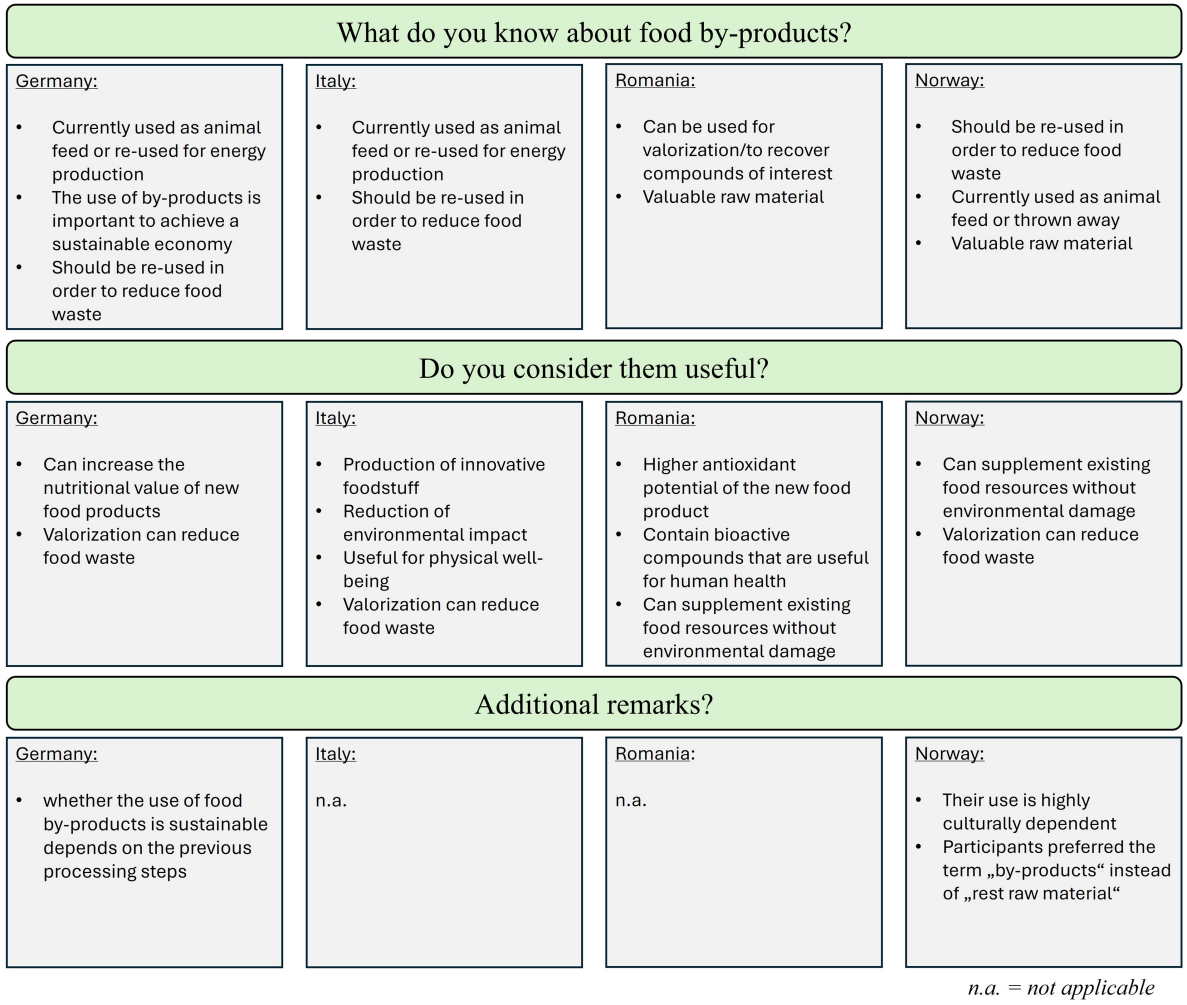
Summary of the major discussion findings of Stage 1: exploration of the general knowledge on food by-products.

The focus group discussion started with a general discussion on by-products to determine the participants’ general point of view and knowledge. In general, we observed an already good knowledge on by-products and the understanding of the importance of their valorization in all four countries. Here, approval of sustainability measures and concerns about the on-going climate change were themes that were identified in all four focus groups without the moderators’ influence. In addition, some participants already proposed ways on how to use these products to create a more sustainable environment, which suggested a general willingness and commitment to create and support a more sustainable economy. The main findings of this section are summarized in Figure 2.

In summary, participants of all groups correctly associated food by-products with food waste or knew about its use as animal feed or energy production. Participants were also familiar with the concept of valorization and shared the opinion that by-products can be a valuable raw material and should therefore be re-used, which again underlined the themes identified above.

When asked for the usefulness of by-products, a reduction of the environmental impact and food waste in general were discussed in each group and positively evaluated. In addition, a new theme could be identified at this point of the study, which was “health aspects” as participants mentioned health benefits through valorization. In detail, the following exemplary statements were made:

- “I agree, they should be used for other products to reduce food waste, but also to increase the nutritional value of products as the obesity rate is increasing” (Germany)
- “I think proteins are one nutrient group that can be easily added to increase the nutritional value” (Germany)
- “As already mentioned, they do often contain many valuable compounds” (Germany)
- “Some by-products can still contain beneficial compounds for humans, but I think it also depends on the previous processing steps” (Italy)
- “I think by-products are important for human health” (Italy)
- “I think the use of by-products is important to produce added value foodstuff. They can be alternatives to natural ingredients” (Italy)
- “Food by-products may contain important compounds for human health” (Romania)
- “These food by-products are useful to obtain products with improved nutritional value” (Romania)
- “Food by-products increase the nutritional value and antioxidant potential of the food product, containing bioactive compounds useful for human health” (Romania)

### 3.3 Stage 2: exploration of food products with by-products

In the second part of the focus group interviews, the benefits of food products containing by-products were evaluated by asking specifically about food products with by-products, and why people could want or avoid them.

During discussions, the participants of all four groups identified a variety of benefits and drawbacks from using by-products as food ingredients in new food products. A list of the identified topics in each country is shown in Table 2.

**Table 2:** Identified benefits and drawbacks of using food by-products as new food ingredients in four different countries.

Country	Benefits	Drawbacks
Germany	Increased nutritional value from health promoting compounds, especially for vegetarians and vegans Recycling of already existing resources that are otherwise wasted	No real waste reduction when only target compounds are extracted Processing is necessary Possibility of new food intolerances/allergies Changes in texture, taste and color Strong marketing necessary
Italy	Reduction of waste and environmental impacts Increased nutritional value of new products Promotion of a circular economy Economic savings	Potential presence of harmful substances Expensive extraction techniques Decreased sensory characteristics
Romania	Ensuring a high intake of target bioactive compounds/ingredients Diversification of the range of food products/nutritionally superior products Minimizing food waste	Emergence of intolerances/allergies to the compounds contained by by-products Complex technological processes, longer processing time Possible consumer reluctance toward unfamiliar concepts Organoleptic changes Separate logistics needed
Norway	Minimizing food waste New food products Contain valuable ingredients	Accumulation of toxins and pollutants Concerns about highly processed ingredients

The identified advantages and disadvantages of using by-products as food ingredients were comparable in all four countries. Main advantages for using by-products as new food ingredients were the reduction of food waste and an increased nutritional value of the newly developed products, which again fits to the themes identified in Stage 1 of the study (see chapter 3.2).

However, during the four discussion rounds, it became obvious that there are currently also many fears and reservations regarding this topic. The major drawback that was identified as theme in all four countries and stated by almost all participants was the topic of food safety. Some statements regarding food safety were:

- “As drawback I can imagine that there will be new food intolerances/allergies” (Germany)
- “I am also afraid of new intolerances” (Germany)
- “I can imagine the presence of dangerous chemical molecules and a high environmental impact” (Italy)
- “I can imagine the presence of dangerous chemical molecules” (Italy)
- “The emergence of intolerances to the compounds contained by by-products” (Romania)
- “Concerned about the levels of pollutants and insecticides” (Norway)
- “food regulations are stricter in Norway than in other countries, therefore it is safer to use the by-products” (Norway)

As minor drawbacks the “impact on taste and texture”/“problems with appearance and taste”/“organoleptic changes,” “adding these by-products to already high processed products” and “using expensive extraction techniques”/“complex technological processes” were mentioned.

In Germany, it was also mentioned that a strong marketing might be necessary to advertise the health benefits of these new products as consumers often stick to the products they already know and are sceptical against new production techniques. In addition, a suggestion from Norway was to choose a positive term for marketing or dissemination activities, as the term “food waste” or “rest raw material” might cause reservations among the consumers, while the term “food by-product” creates a neutral or positive association.

### 3.4 Stage 3: exploration of purchases and consumption trends & decision-making processes

Furthermore, the participants were prompted to write down examples of food products with or from by-products, and to name products they would like to buy (multiple answers were possible). In addition, participants were also asked for their reasons and in which situations they would most likely consume these products.

**Figure 3:**
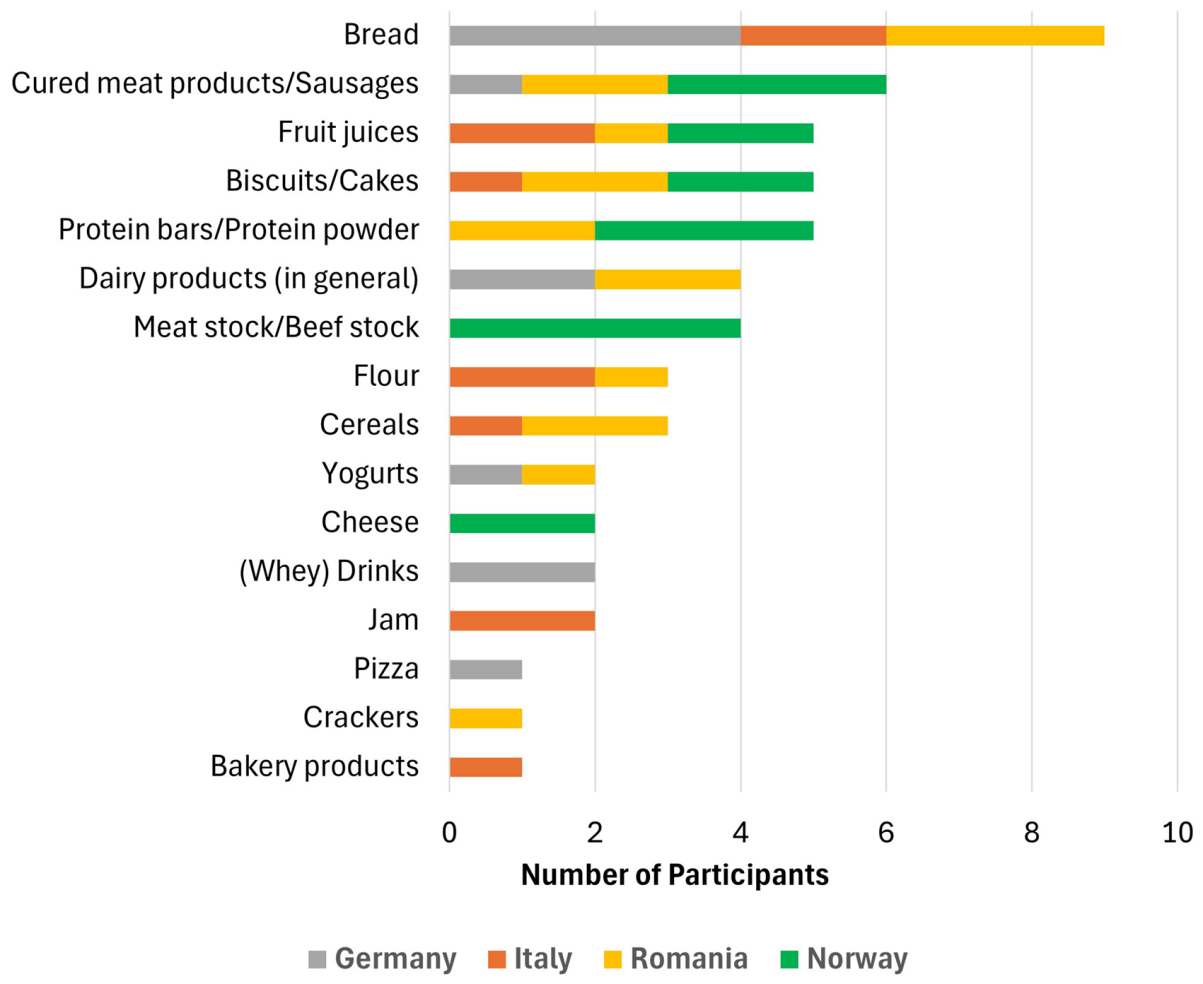
Summary of food products supplemented with ingredients from by-products that participants would prefer to buy.

As no pre-formulated answers were given, the results varied and covered a broad area of products (see Figure 3). Nevertheless, protein- or fiber-enriched bread was named by several participants from all countries except Norway, where the main type of bread consumed is already full-grain and consequently fiber-rich bread. Along with bread, bakery products in general seemed to be favorable items for adding food by-products as there were also listings of biscuits and cakes, pizza, crackers, and simple flour that can be used for baking. There was also an interest in dairy products, especially yogurts, cheeses and milk-based drinks, or dairy products in general that can be enriched with whey proteins (for example). In addition, fruit-based products like fruit juices and jams were also mentioned by a few participants, which might be interesting when valorizing by-products from the fruit industry, like peels and seeds. Interestingly, cured meat products/sausages, as well as meat stock were the second most preferred products.

When asked for the reason behind their decision, answers did not differ a lot between the four countries. Some examples were:

- “Longer feeling of satiety, healthy image” (Germany)
- “Bread is a staple food for me, adding more nutrients to food is good and necessary” (Germany)
- “Improved digestion, beneficial ingredients” (Italy)
- “Keeps me full for a long time and it’s healthy” (Italy)
- “Higher content in fibers, proteins, essential fatty acids and compounds with antioxidant activity” (Romania)
- “I eat bread daily and I consider it a necessity. It is better if the fiber content can be increased” (Romania)
- “They taste good” (Norway)
- “They are healthier/contain something that the diet has too little of” (Norway)

In summary, it became obvious that the majority of participants perceives these products as healthy due to increased fiber or protein contents. The participants also expect benefits for their digestion system and their overall wellbeing due to the beneficial ingredients without taking supplements. In addition, having a longer feeling of satiety due to increased protein and fiber contents was also mentioned several times. Another important aspect was that the mentioned products are compatible with their diet and/or that they already consume products like that and would like them to be more nutritious. However, one participant from Norway and one from Germany mentioned that they “prefer less processed foods” or at least “want to know how the product was processed”.

In the final part of the focus group study, the participants were introduced to the general idea of enriching products using by-products from different food production chains, such as dairy, oilseeds, brewery, meat (poultry) and the prickly pear cactus. Afterwards they were asked to write down which by-products they would prefer to eat and what the reasons for their decision are. For this decision-based question, participants were allowed to give multiple answers.

In Germany, the three by-products of choice that were almost equally mentioned were oleaginous by-products, dairy and brewery by-products. When asked for their reasons behind this decision, the participants choosing brewery by-products stated that these contain reasonable amounts of both fiber and proteins and that the brewery industry is quite big in Germany. Consequently, it makes sense to use this material due to the available amounts and the regional availability. One participant chose them because she is vegan and “spent grains contain the amino acid lysine, which is important for vegans” and she likes that to be added in food that she regularly consumes. The participants that have chosen dairy by-products explained that they like the taste of whey and that whey has many beneficial ingredients. Reasons for choosing oleaginous seeds were the beneficial ingredients and that it can easily be integrated into bread, which is a staple food in Germany.

In Italy, by-products from the oilseed industry (oleaginous seeds) were mentioned by every participant, and 25% of all votes were for by-products from the dairy industry, while brewery and cactus by-products were only listed by a few participants. Poultry by-products were not mentioned at all. The main reasons for their decision were: the traceability and safety of the chosen by-product can easily be monitored (4 out of 6 participants), safety and ethical reasons (1 participant) and nutritional properties (1 participant).

In Romania, 4 participants have chosen oleaginous seed by-products and 2 participants voted for dairy by-products. Reasons for the oilseeds were their nutritional benefits, e.g., high fiber content, polyphenols and anti-inflammatory properties, as well as the ease of integration of this by-product type into new products. The participants that preferred the dairy by-products stated that they already consume similar products and that dairy by-products have both taste and nutritional properties.

In Norway, participants were open to four out of the five presented by-products. When asked for the reasons behind their decision, most participants stated the importance of taste and that they are already familiar with their chosen by-product type. One participant chose his by-product due to its high fiber content. Interestingly, three participants were interested in cactus by-products even though they have never tasted anything made out of cactus before.

### 3.5 Study limitations

Focus group interviews are a good fit for a study like the one presented here, however, some drawbacks could be identified due to the general structure of a focus group interview: first, participation needs to be optional at all times. Without financial compensation or other benefits, only people interested in the specific topic will be willing to invest their time in a study like this, which means that they are often more educated in the area of the study topic than others. In addition, the recommendation that focus groups should consist of 6–8 participants can be problematic as well. Although this small group size has many benefits as mentioned above, it can be hard to truly represent a certain population group. Especially in the study presented here, it was not always possible to form groups that reflect the general population due to the broad age range, but also due to the variety of other parameter that needed to be investigated, like food preferences and educational level. To obtain more in-depth results, this study could be repeated with differently structured groups, e.g., one vegetarian/vegan group, groups with a smaller age gap, female and male only groups and groups with different educational backgrounds. For this study, however, this approach would have been too time consuming and almost impossible to implement, especially without compensating participants for the study time. But still, the presented approach was suitable for our intended goal, which was to get a first insight into consumers’ interest for new food products from by-products in different countries.

## 4 Discussion

In recent years, many studies like ours were conducted in the field of consumer acceptance of new food products. While most of them targeted foods or ingredients like insects, cultivated and 3D-printed meat, also some investigations on upcycled foods made from by-products were previously conducted (20–23). All those studies have in common that so-called novel foods are the focus of interest, as they have often shown a reduced acceptability in the past.

In general, novel foods cause many reservations among consumers with food (technology) neophobia being the most often identified drawback. According to Monaco et al. (24) this can partly be explained by the decision from the EU to consider all products not consumed before 1997 as novel foods, and the authorization procedure that all novel foods have to pass before market implementation (25). However, also environmental concerns, disgust and fear, a perceived artificiality of novel foods as well as unpleasant or unfamiliar sensory attributes were identified to decrease the consumer acceptance in the past, also when talking about upcycled foods from by-products (20, 23, 24, 26, 27).

With by-products in particular, Lu et al. (28) identified three categories of key factors when regarding the consumers’ acceptance: sociographic characteristics, psychographic characteristics and product characteristics. In their study, they suggested that there is an impact on acceptance of upcycled foods by gender, educational level, food (technology) neophobia, environmental awareness, food waste awareness, communication about health benefits, sustainability, food waste reduction and economy. Here, McCarthy et al. (29) and McCarthy et al. (30) have observed that consumer with a greater awareness of food waste were more open to buying upcycled foods made from fruit and vegetable waste when compared to others with a lower awareness of food waste problems. This was also found in a study by Altintzoglou et al. (31), where people showed a higher positive attitude toward new foods from seafood by-products after getting information on the positive contribution to public health and to food waste reduction compared to the consumers, who received only a general definition. This is in agreement with the results from Aschemann-Witzel et al. (32) where environmentally concerned participants showed a higher interest in upcycled bakery, dairy and snack products. In a different study from Perito et al. (33, 34), consumers believed that upcycled food products have a low environmental impact, which could also be observed in our study. It was also shown that sustainability aspects of enriched and upcycled foods positively influence the willingness to purchase those goods (33), however, participants in our focus group studies additionally stated that those products should not be more expensive than the regular ones.

While the background knowledge of foods made with by-products was high in our study, several other authors identified a knowledge gap among their participants when talking about upcycled foods. In a study from New Zealand, only 10% of participants were familiar with this topic (35), while 85% of British consumers were not familiar with the upcycling of food as well (22). In Turkey, the percentage was with 35% slightly higher, but it was highlighted that 83% of those who were familiar with food upcycling had a higher education, i.e., a bachelor’s degree or higher (36). This finding can also explain the high knowledge among our focus group participants, as all participants were either university students or held at least a bachelor’s degree. In addition, Italians were also found to be familiar with the concept of upcycling before as in a previous study only 19% of participants have never heard of this topic before (37).

Besides knowledge on food waste and upcycled foods, some other strategies and points of attentions could be identified in previous studies, which are also in line with our results. First, it was shown that well-known products might increase consumer acceptance (26, 37, 38). This was also our observation when asking the participants on food products they would like to consume. Here, many of them explained their choice by stating that they already consume products like that, which means that they can be easily integrated into their diet. The acceptance, however, will only be given if the appearance and taste is similar to the well-known taste, which was also mentioned by several participants of our study. Here, food industries must carefully proportionate the amount of novel ingredients, in our case novel ingredients from food by-products, to not alter the sensorial properties of the product (22, 27, 37, 39). Besides that, we identified communication as one of the key factors for implementing new food ingredients or food products. While this is something the participants also mentioned themselves, we also observed that many of them were more open to the concept of upcycled food after explaining the benefits of enriched products. Communication was also found previously to be the most important strategy for increasing consumer acceptance of foods with new ingredients (12). Here, especially communication about the health benefits of enriched foods has shown to be able to increase the consumers’ acceptance. But also communication of environmental benefits, scientific findings and ethical issues was found to be equally important. Targeted and effective communication strategies should be used to increase the consumers’ awareness on certain topics and to reduce their scepticism about new green technologies (23, 40–43). In addition, cooking recipes with the new food ingredients or food products might help the consumers to implement these into their diets (44, 45). Among others, Lu et al. (28) emphasized that the inclusion of logos, labels and certifications on the product package are important decision points as well (20, 28, 36, 37, 46, 47). Here, nutritional information, health claims, ingredient lists, allergen information, and labels related to sustainability and ethical considerations are the main decision-drivers of consumers. However it is important to be aware of that even if increasing how transparent the communication about the sustainability of the food product is, this does not necessarily lead to improved consumer perception of the food product (48). This makes communication programs on how to interpret food labels rather important as not every consumer may fully understand the provided information, which may lead to misinterpretation (20, 49–51).

## 5 Conclusion

The main objective of this study was to obtain qualitative information through a series of focus group interviews with consumers in selected countries, namely Germany, Italy, Romania, and Norway, and to generate input for new product development of foods with by-products.

In general, the participants from all four focus groups have already heard of the concept of valorizing by-products and also of food products containing by-products. Associations with valorizing by-products were positive as they are seen as source of healthy compounds, while at the same time food waste can be lowered by valorization, which is beneficial for the environment. Participants in all four countries were interested especially in bakery and meat products as well as protein powders and dairy products. The main reasons for their decision were that they are already familiar with these foods and would like them to be more nutritious.

However, as the aspect of food safety was one of the major concerns from the participants in our study, this topic should be researched intensively and disseminated among the general public, not only researchers. In general, we found that scientific communication might be the most useful way to promote new ways of (green) food production and to allow a successful market implementation of food products made from by-products.

## Data Availability

The original contributions presented in the study are included in the article/Supplementary material, further inquiries can be directed to the corresponding authors.
