# Supplementary material for: Consumers acceptance of new food ingredients from the food industry’s by-products—a focus group study
Source: Front Nutr. 2025 Mar 19;12:1509833. doi: 10.3389/fnut.2025.1509833 (PMC11961439; doi:10.3389/fnut.2025.1509833)
Supplement: Supplementary file 1 [file Table_1.docx]

Supplementary Material

# Supplementary Material 1: Questionnaire for Participant Selection

Contact information:

Name: _______________________________________

e-mail: _______________________________________

Telephone number: _____________________________

Q1. What is your occupation? Probe for nature of job and industry

|  |
| --- |

**CLOSE IF ANY MENTION OF OCCUPATIONS IN THE BELOW TABLE**

| □ Food/ Drink | 1 | Close |
| --- | --- | --- |
| □ Retail | 2 | Close |
| □ Market research | 3 | Close |
| □ Advertising | 4 | Close |

**MESSAGE: “Unfortunately you fall outside our target group for this study. Thank you for your time.”**

Q2a. Have you attended any food-related market research events in the past 6 months?

| □ Yes | 1 | Close |
| --- | --- | --- |
| □ No | 2 | Skip to Q3 |

**CLOSE IF YES.**

**MESSAGE: “Unfortunately you fall outside our target group for this study. Thank you for your time.”**

Q3. Are you the main decision maker with regards to grocery shopping?

| □ I’m the main decision maker | 1 | Continue |
| --- | --- | --- |
| □ I am the joint decision maker alongside other family member | 2 | Continue |
| □ Someone else in my family is main decision maker | 3 | Close |

**CLOSE IF NOT MAIN OR JOINT DECISION MAKER WITH REGARDS TO GROCERY SHOPPING**

**MESSAGE: “Unfortunately you fall outside our target group for this study. Thank you for your time.”**

Q4. Which of the following items do you consume?

|  | Once a week or more | 2 -3 times a month | Once a month | Rarely than once per month | Never |
| --- | --- | --- | --- | --- | --- |
| Whey products (cheeses, butter, protein concentrate, whey powder etc.) | □ | □ | □ | □ | □ |
| Whole Cereals | □ | □ | □ | □ | □ |
| Oleaginous seeds | □ | □ | □ | □ | □ |
| Bread from different cereals/oleaginous seeds | □ | □ | □ | □ | □ |
| Foods containing prickly pear cactus by-products or ingredients extracted from prickly pear cactus by-products | □ | □ | □ | □ | □ |
| Yogurts with increased protein content | □ | □ | □ | □ | □ |
| Milk | □ | □ | □ | □ | □ |
| Bread with increased fiber content | □ | □ | □ | □ | □ |
| Poultry by-products (liver, kidney, brain etc.), or foods containing poultry by-products or ingredients extracted from them | □ | □ | □ | □ | □ |

Q5. Tick the products that contain protein:

| □ Yogurt | 1 |  |
| --- | --- | --- |
| □ Oatmeal (dry oats) | 2 |  |
| □ Meat | 3 |  |
| □ Sugar | 4 |  |
| □ Milk | 5 |  |

**IF PARTICIPANTS TICK SUGAR TO CONTAIN PROTEIN CLOSE THE QUESTIONNAIRE**

**MESSAGE: “Unfortunately you fall outside our target group for this study. Thank you for your time.”**

Q6. Gender:

| □ Female | 1 |  |
| --- | --- | --- |
| □ Male | 2 |  |

**AIM FOR MALE / FEMALE 50% /50%**

Q7. Can you please tell me your age (record specific age): ………………………………

| □ Below 30 | 1 |  |
| --- | --- | --- |
| □ 30-40 | 2 |  |
| □ 40-50 | 3 |  |
| □ 50-60 | 4 |  |
| □ above 60 | 5 |  |

**GOOD SPREAD OF AGES FOR THE ‘GENERAL POPULATION GROUP’**

Q8. What is your marital status?

| □ Single, at parental home | 1 |  |
| --- | --- | --- |
| □ Single, living independently | 2 |  |
| □ Married / Co-habiting | 3 |  |
| □ Separated / Divorced | 4 |  |
| □ Other (Specify)____________________________________________ | 5 |  |

Q9a. Have you got any children living with you?

| □ Yes, I have children living with me | 1 | Go to Q9b |
| --- | --- | --- |
| □ No, I do not have children living with me | 2 | Go to Q10 |

Q9b. How many children do you have?

|  |
| --- |

Q10. What is your level of education?

| □ Secondary school without qualifications | 1 |  |
| --- | --- | --- |
| □ High school or vocational training | 2 |  |
| □ Bachelor degree | 3 |  |
| □ Master degree or PhD | 4 |  |

Q11. When is more convenient for you to participate in the discussion:

| □ Monday, ……………… at. 16.30-18.30pm |
| --- |
| □ Tuesday, ……………….at 16.30-18.30pm |

# Supplementary Material 2: Moderators’ Protocol

Template for **Stage 1: Exploration of general knowledge on food by-products**

| **Participant Name**  **(Focus group - general population** | *What do you know about food by-products? Are these important? Can these be valorised? Do they contain important compounds for humans health? Do you consider that they are useful? For what?* |
| --- | --- |
|  |  |
|  |  |
|  |  |
|  |  |
|  |  |
|  |  |

Template for **Stage 2: Exploration of food products with by-products**

| **Participant Name**  **(Focus group - general population** | *Have you heard about food products containing by-products or from by-products?* |
| --- | --- |
|  |  |
|  |  |
|  |  |
|  |  |
|  |  |
|  |  |
| **Name** | *Why would somebody use (want to use) or avoid to use by-products ?* |
|  |  |
|  |  |
|  |  |
|  |  |
|  |  |
|  |  |
| **Name** | *What are benefits and drawbacks could you expect from adding by-products in new foods or valorising by-products for extracting other ingredients ?* |
|  |  |
|  |  |
|  |  |
|  |  |
|  |  |
|  |  |

Template for Stage 3

| **Focus group - general population** | | | |  |  |
| --- | --- | --- | --- | --- | --- |
| **Participant Name** | | | |  |  |
| *Write down all the examples that come to your mind of type of food products containing by-products?* | *Write down all the examples that come to your mind of type of compounds/ingredients extracted from by-products?* | *Rank products you would prefer to buy (1-the most likely to buy)* | *Additional information for products ranked with 1:*  *Why did you prefer these ones?* ***(those most likely to buy)***  *What other reasons could you have for their preference?* | *Rank products you would prefer to consume (1-the most likely to consume)* | *Additional information for products ranked with 1:*  *Does specified product have a special benefit in that consumption situation?* ***(those most likely to consume)***  *Why would participant consume it? (Why would you like to consume product with/from by-products in that situation?)* |
|  |  |  |  |  |  |
|  |  |  |  |  |  |
|  |  |  |  |  |  |
|  |  |  |  |  |  |
|  |  |  |  |  |  |
|  |  |  |  |  |  |

Moderator will read this information to the participants and explain the benefits of this kind of products.

- Brewer spent grain (BSG) are a major by-product from beer production currently used as low-value animal feed. General BSG constituents are 30-50% fibres and 20-30% proteins, the latter containing valuable essential amino acids like lysine. Fibres like arabinoxylans and β-glucan are beneficial for gut health and show cholesterol-lowering effects.
- Biomass from oilseeds by-products is a very relevant source of phytochemicals; sunflower seeds and sprouts show antioxidant, antimicrobial, anti-inflammatory and antihypertensive properties thanks to phenols, flavonoids, polyunsaturated fatty acids and vitamins. The protein content is high (> 36% proteins, it contains relevant amounts of dietary fiber, vitamins and minerals.
- The production of chicken meat generates high amounts of by-products. A large part of these byproducts is utilized, but a significant portion of meat is still left on the bones after filleting and production of mechanically deboned meat. These proteins can be extracted using enzymatic hydrolysis.
- Main generated by-products and wastes in dairy industry are whey, dairy sludge and wastewater, all characterised by high nutrient levels.
- There is a growing demand for cactus cladodes consumption due to the nutritional and functional value. The most promising constituent is mucilage, a hetero-polysaccharide with gelling properties and antihyperlipidemic, antiobesity and hypocholesterolemic effects.

Thus, participants will be asked **to imagine that they want and need to consume** increased protein/fibers as a protein/fibers supplement (e.g., because of protein deficiency, digestive difficulties) and **to think of a product they would prefer to consume as a protein supplement that could involve product with proteins/fibers** anything from yoghurts, snacks, breads, biscuits, pasta or vegetable / meat alternatives (meaning types of products like soy meat, vegetable patties etc**.).** Hence, they will be prompted with the question:

- *Would you prefer these products to be supplemented with by-products from dairy, oilseeds, brewery, meat (poultry), prickly pear cactus?*
- *Specify which type of by-products would you prefer? (ask only if they did not mention it)*
- *Could you elaborate reasons for your preference / opinion?*

| Focus group |  |  |  |  |  |
| --- | --- | --- | --- | --- | --- |
| Participant name |  |  |  |  |  |
|  |  |  |  |  |  |
|  |  |  |  |  |  |
|  |  |  |  |  |  |
